# Fake publications in biomedical science: red-flagging method indicates mass production

**DOI:** 10.1007/s00210-025-04275-9

**Published:** 2025-09-24

**Authors:** Bernhard A. Sabel, Emely Knaack, Gerd Gigerenzer, Mirela-Ioana Bilc

**Affiliations:** 1 Institute of Medical Psychology, Medical Faculty, Otto-Von-Guericke University of Magdeburg, Leipziger Straße 44, 39120 Magdeburg, Germany; 2 Center for Brain and Behavioral Sciences (CBBS), Magdeburg, Germany; 3 Max-Planck Institute for Human Development, Berlin, Germany

**Keywords:** Biomedical science, Paper mill, Research integrity, Fake, Trust, Science fraud

## Abstract

Integrity of academic publishing is increasingly undermined by fake publications massively produced by commercial “editing services” (so-called “paper mills”). These services use AI-supported production techniques at scale and sell fake publications to students, scientists, and physicians under pressure to advance their careers. Because the scale of fake publications in biomedicine is unknown, we developed an easy-to-apply rule to red-flag potentially fake publications and estimate their number. After analyzing questionnaires sent to authors of published papers, we developed simple classification rules and tested them in a 9-step bibliometric analysis in a sample of 17,120 publications listed in PubMed®. We first validated various simple rules and finally applied a multifactorial tallying rule comparing 400 known fakes with 400 random (presumed) non-fakes. This rule was then applied to 1,000 random publications each from 2020 and 2023. The multifactorial tallying rule was the best red-flagging method, with a 94% sensitivity and only a 11.5% false-alarm rate. The rate of red-flagged articles increased during the last decade, reaching an estimated 14.9% in 2020 and 16.3% in 2023. Countries with the highest proportion of read-flagged publications were China, India, Iran, Russia, and Turkey, with China and India the largest absolute contributors globally. Applying Bayes’ rule resulted in an estimate of 5.8% actual fakes in the biomedical literature. Given 1.86 million Scimago-listed biomedical publications in 2023, we estimate the actual number of true fakes at 107.800 articles per year, growing steadily. Scientific publications in biomedicine can be red-flagged as potentially fake using fast-and-frugal classification rules to earmark them for subsequent scrutiny. When applying Bayes´rule, the annual true scale of fake publishing in biomedicine is about 19 times that of the 5.671 biomedicine retractions in 2023. This scale of fraudulent publishing is concerning as it can damage trust in science, endanger public health, and impact economic spending. But fake detection tools can enable retractions of fake publications at scale and help prevent further damage to the permanent scientific record.

## Introduction

Trust in the integrity of academic publishing is a foundation of science, and lack of it damages its reputation (Behl 2021; Byrne 2019; Seifert 2021; Else and Van Noorden 2021; Else 2022; Byrne et al. 2022). Well-known cases of scientific misconduct by individual researchers include ghost and honorary authorships (Flanagin et al. 1998; Wislar et al. 2011; Frederickson and Herzog 2021), cherry-picking, abstract spin, plagiarism of images (Bik et al. 2016), and outright data fabrication (Bik 2020; Byrne and Christopher 2020; Park et al. 2022), and some of these publications were retracted (Table 1). Although individual fraud has been recognized for centuries, the recent emergence of commercial production of fake publications is a new and unprecedented development (Flanagin et al 1998; Wislar et al. 2011; Mavrogenis et al. 2018; Byrne 2019; Byrne and Christopher 2020; Else and Van Norden 2021; Sabel and Seifert 2021; Chawla 2022; Candal-Pedreira 2022). The major source of fake publications are 1,000 + “academic support” agencies—so-called paper mills—located mainly in China, India, Russia, UK, and USA (Abalkina 2021; Else 2021; Pérez-Neri et al. 2022; consider Wittau and Seifert 2024 for a comprehensive review). Paper mills openly advertise writing and editing services on the internet and charge hefty fees to produce and publish fake articles in journals listed in the Science Citation Index (SCI) (Christopher 2021; Else 2022). Their services include manuscript production based on fabricated data, figures, tables, and text semi-automatically generated using artificial intelligence (AI). Manuscripts are subsequently edited by scientifically trained professionals and ghostwriters. Although their quality was initially relatively low (Cabanac and Labbé, 2021), fake publications are now more sophisticated and can easily pass peer review in established journals (Seifert 2021). Of note, leading publishers profit indirectly from the paper mill business model by collecting open access fees for fake articles, and authors and paper mills are even gaming (manipulating) the journal impact factor (JIF) metrics. Though some governments, funding bodies, and academic publishers are now on the alert (Abalkina et al. 2025; Brainard 2023; Candal-Pedreira 2022; Cyranoski 2018; Else 2022; Mallapaty 2020; Sabel 2022; Van Noorden 2023), many scientists, journal editors, learned societies, and funders appear to be surprisingly unaware of the industrial scale of paper mill activities.

**Table 1:** Number of known fake publications in the biomedical literature

Published reports of known fake publications			
Reference	Years analyzed	No. of fakes	Type of analysis
Heck et al. (2021)	2021	75	Image forensics, raw data requests
Seifert (2021)	2019–2020	100	Raw data requests
Mallapaty (2020)	2017	107	Retraction analysis
Behl (2021)	2017–2021	137	Image forensics
Elango (2021)	1992–2020	161	Retraction analysis
Lei and Zhang (2018)	1997–2016	260	Retraction analysis
Abalkina (2021)	2019–2021	434	Offers from paper mill website
Bik (2020)	2016–2020	633	Image forensics
Bik et al. (2016)	1995–2014	784	Image forensics
Brown et al. (2022)	2019	1,396	Retraction analysis
Fang et al. (2012)	2010	1,611	Retraction analysis
Candal-Pedreira	2022	3,544	Retraction analysis
Ataie-Ashtiani (2018)	2011–2017	4,931	Retraction analysis
RetractionWatch	All until 2024	> 50,000	Different reasons (approx. 1/3 are presumably “honest” mistakes)

Selection of prior studies that quantified the number of fake publications in the years 1982–2024 as identified by text or image plagiarism, data fabrication, paper mill offers, and retractions. In 2023 alone, more than 10,000 papers were retracted (Note: duplicate counts could not be determined). Most retractions originate from China, Pakistan, Russia, and Saudi Arabia (96.8% of retractions on account of paper mills are from China; Candal-Pedreira, 2022; retractions listed at https://retractionwatch.com/*).* Given the 61 million publications in all science disciplines and all years (https://www.scimagojr.com/worldreport.php), the percentage of roughly 50,000 known fakes (retractions) is 0.00082%.

Paper mill customers—students, physicians, and scientists—experience pressure by incentive systems of their academic or government institutions or university-affiliated hospitals to publish in SCI journals (Pérez-Neri et al. 2022). For example, the Beijing municipal health authorities require a fixed number of first-authored SCI articles for physicians to qualify for promotion (Else and Van Noorden 2021). The pressures of the “publish or perish” culture are enhanced by academic policies that count publications and value journal impact factor (JIF) metrics as surrogates for scientific excellence and make salary increases, promotion, or scientific reputation dependent on these which varies among disciplines, institutions, and national policies (Van Dalen and Henkens 2012; Lim et al. 2025). Paper mills offer their services to accomplish these goals. They do not openly advertise their fraudulent production but instead camouflage themselves as academic editing or ghostwriting agencies ready to “help prepare” masters or doctoral theses and publications, and their number is on the rise (Abalkina et al. 2025). Our Google and Waibo search found over 1500+ such agencies. Of course, some may be authentic editing services that check for language errors, but others are not.

We are aware of several instances where paper mills tried to promote their business by inviting and bribing journal editors to collaborate. For instance, in his role as the editor of a biomedical journal, the first author (BAS) received this unsolicited email in 2022 from a paper mill (see also Table 2):

“We are a well-known academic support institution from Guangzhou, China, which has been established for 8 years. … For reducing the publication time, we expect to cooperate with you in the future. Cooperation mode: we cite the content of your journal in our articles, thus increasing … your impact factor in 2022. You shall help us shorten the publication time. Payment: If an article is successfully published, we will pay for it at the price: IF*1,000 USD/article. For example, with IF=2.36, total payment=2.36*1,000 USD=2,360 USD. And this price is negotiable”.

**Table 2:** Rendezvous with a paper mill—a true story

An unsolicited email from a paper mill to an SCI journal editor, and the following exchange (January-April 2022). The editor followed up the contact per email and recorded a Zoom meeting to detail the fraudulent business model, including evidence of corruption. **Paper Mill** We are a well-known academic support institution from Guangzhou, China, which has been established for 8 years. Experts in our institution need to publish some research papers in SCI journals every month. For reducing the publication time, we expect to cooperate with you in the future. Cooperation mode: we cite the content of your journal in our articles, thus increasing … your i in 2022. You shall help us shorten the publication time. Payment: If an article is successfully published, we will pay for it at the price: IF*1,000 USD/article. For example, with IF = 2.36, total payment = 2.36*1,000 USD = 2,360USD. And this price is negotiable What you can get: 1. An increased journal impact factor in 2022 2. Desirable payment we give **Editor** [Journals] are always happy to receive manuscripts for publication and always welcome suggestions of how to increase paper flow. …I have a few questions … so that I can better understand what to expect: 1. How many scientists/publications have you supported and in which fields do you have experience? How about the field of medicine, especially neurology? 2. How can I be sure that the quality of the manuscripts is good enough for our journal? Do you have foreign native speakers who check the language thoroughly? 3. How many manuscripts can I count on receiving from you per month? Is the number large enough to reach a noticeable growth of my journal in a relatively short period of time? 4. You mentioned a method to increase the impact factor. Can you explain how that works and what impact factor growth you can accomplish….? ….We are currently at around IF 2. How long would it take … to reach IF 5 or 6…? **Paper Mill** 1. We have supported the publication of thousands of articles. We mainly have experience in biomedical fields, such as molecular biology, pharmacy, and tumor-related research 2. We have people from native English-speaking countries to polish the manuscript and experts with biomedical background to review the content. We will arrange 3–4 colleagues to work with you 3. We have more than 100 manuscripts every month. … 4. We are very familiar with a series of ethical norms of academic publishing, and we also know that the influencing factors of a magazine are related to the number of manuscripts published and the number of times articles are cited by other scholars. Therefore, we will vigorously publicize and recommend the articles of the magazine to Chinese academics on our own publicity platform. Get maximum exposure. … **Editor** 1. We are planning a special issue on “Neuromodulation… for neurorehabilitation.” We are wondering if you are able to send us 8–12 manuscripts on the topic within the next 4–6 weeks … 2. These days it is sometimes difficult to find qualified reviewers that are familiar with this field. Therefore, for each paper, would you be able to suggest at least 3 independent researchers knowledgeable in this field … willing to review the manuscript? … 3. To estimate the growth potential of any collaboration: How many full-time employees does your agency employ? And do you have any access to freelancers, and if so, from which countries (language)? 4. Do you have any unique advantage over other agencies that have similar offers? 5. In order to evaluate the quality of your service, could you please send us … references of SCI-papers … you have helped to get published in the field of medicine? **Paper mill** 1. We have our own molecular biology laboratory and have developed extensive cooperation with many universities in China. Therefore, we think we can send sufficient manuscripts on this topic 2. First, we have colleagues who have worked at MDPI, AME Publishing Company, Hindawi, and so on, and they are responsible for suggesting qualified reviewers. Second, our company has many alumni from Sun Yat-sen University and the University of Hong Kong, and they will review our manuscripts more friendly [sic] 3. Our company currently has 100 full-time employees and more than 300 freelancers from the United States, Canada, Italy, Israel, Pakistan, Turkey, India, China and other countries 4. Our advantage lies in the stable customer source, as well as the mature and efficient management system 5. Here are the DOIs: …	

Unexpectedly, the paper mill was willing to provide a DOI-list and PDFs of 98 of its publications, 96 of which were red-flagged by our indicators as fake

This attempted corruption motivated us to quantitatively analyze and estimate the global scope of fake publishing. While the relatively small number of retracted publications might suggest that the problem of fake publishing is small (van Noorden 2023), publishers and learned societies have already begun to adjust editorial, peer-review, and publishing procedures and use AI for fake indicator identification and for streamlining their publishing processes. Yet the actual scale of fake publishing remains unknown or is not publicly communicated.

To estimate the scope of fake publishing, we therefore developed a method to red-flag potential fake publications (RFPs) with indicators that are easy to use by reviewers, editors, and publishers and tested their feasibility in randomly selected pubications. As we now show, a screening for potential fakes using a validated detection tallying rule with a high sensitivity and low false-alarm rate revealed an estimated rate of 16.3% red-flagged articles for 2023. Based on this rate and the sensitivity and false-alarm rate of the detection tallying rule, calculating Bayes’ rule results in an estimated annual 5.8% (107.800) of annual true fakes in the biomedical literature.

## Methods

**Fig. 1:**
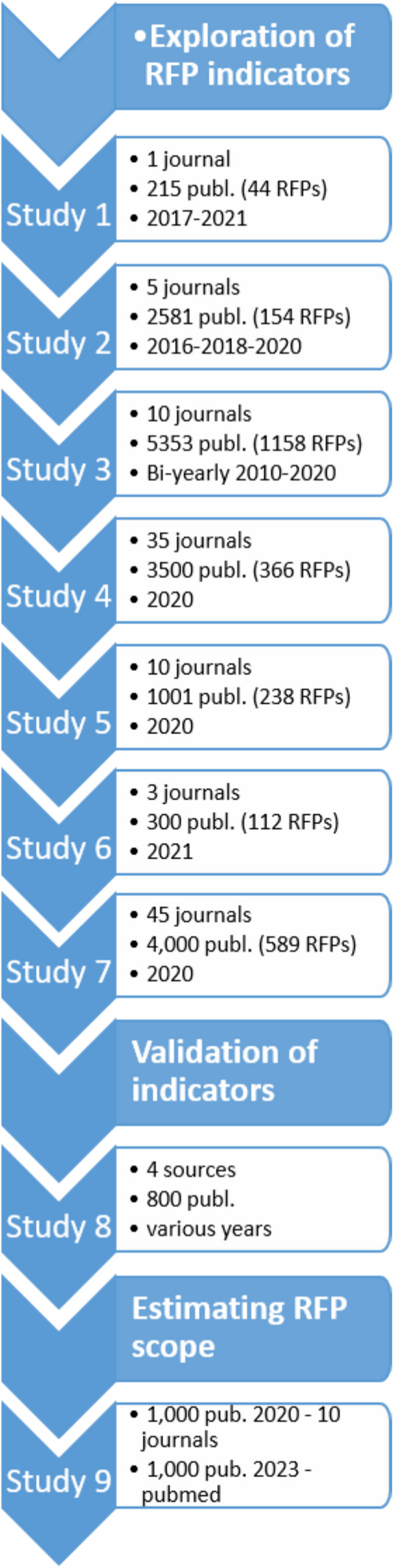
Screening plan for journals and publications. Studies 1–6 identified potential indicators of fake publications on the basis of hypotheses 1 to 3 (see text), using the indicators “private email” and “no international author”, Studies 7 used the indicator combination “email” and “hospital” as an alternative classification rule. To estimate the incidence of potential fake publications in Studies 8 and 9, we used a more complex multifactorial tallying rule. Note that some publications were used in several screening studies. Total number of unique publications analyzed: *n* = 17,120

With a series of 9 bibliographic studies, we tested different indicators in a sample of 17,120 publications listed in PubMed and Web of Science in the fields of neuroscience and medicine (Fig. 1). The present report is an update of our earlier results, which were archived as a preprint (https://www.medrxiv.org/content/10.1101/2023.05.06.23289563v2).

### Exploration

To search for potential fake indicators, in Study 1, questionnaires were sent to corresponding authors of published articles suspected of fraud, and to a control sample of unsuspicious articles (see Table 3). Based on the divergent willingness to respond, we identified potential indicators for fake publications. These indicators should satisfy the following patterns (see below):

(i) Authors of fake publications are reluctant to provide critical information as revealed by their response—or non-response—to the editor’s questionnaire,
(ii) the number of fake publications increases steadily over time, and
(iii) journals with a low to medium impact factor (JIF) are most affected.

**Table 3:** Questionnaire sent to corresponding authors

Questionnaire on “Good Scientific Practice”
Name of first author with ORCID, if available
Name of corresponding author with ORCID, if available, and number of his/her SCI journal publications
Data collection and analysis of the work described in this publication complies with good scientific practice and data and their analysis are real. [Yes/No]
List institutional (!) (professional) email addresses of all co-authors (in the order of authorship)
Did you engage a professional agency to help write your paper? [Yes/No] If yes, please state the agency name, website, and email
If so requested by the editor, are you able and willing to provide the original data and images of your publication? [Yes/No]
Was the manuscript originally submitted simultaneously to other journals? [Yes/No] If yes, specify all journals and the email address of their editorial offices
Provide name and institutional email of your academic leadership: 1. Academic leader (e.g. President/Dean) 2. Head of HR Department
If your work received grant support, please specify the source(s) and provide contact information (name of institution/agency/grant)
Please sign to confirm the following statement: *“I declare that I take the responsibility that the data and content of the above-mentioned publication are authentic and that they comply with the principle of good scientific practice.”*

We observed that publications fulfilling these three criteria shared easy-to-detect fake-indicators: The corresponding author had a private (non-institutional) email address, no international co-author, and/or a hospital affiliation. These features were tested in two simple “AND” rules: “If both indicators are present, classify as a potential fake, otherwise not” (Katsikopoulos et al. 2020). The rule “private email address AND no international coauthor” (for short, email-coauthor rule) was used in Studies 2–6 to flag suspicious publications. Study 2 applied the email-coauthor rule to five neuroscience journals; Study 3 increased sample size to estimate RFP growth from 2010 to 2020, including five journals in the field of general medicine. Studies 4 and 5 increased sample size further, and Study 6 estimated RFP rates in three open access Frontiers journals. After discovering that many red-flagged publications from Studies 1–6 had a hospital affiliation; in Study 7, we applied the AND-rule “email-hospital” to classify 4,000 publications of 45 journals from 2020.

**Fig. 2:**
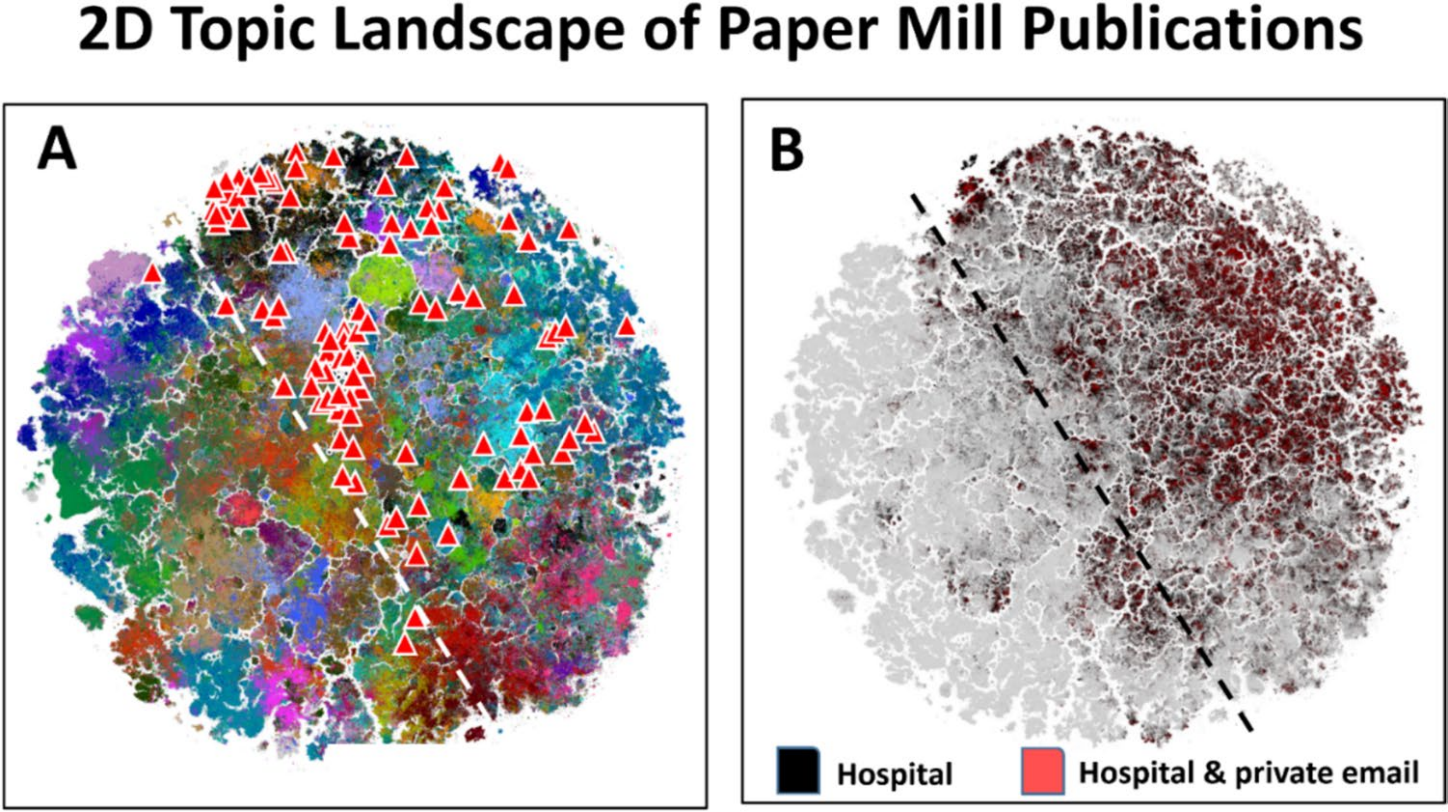
2D landscape map of biomedical topics of 21 million biomedical publications. **A** Color patches represent different disciplines (topics) in basic science to the left of the white line (e.g., immunology, physiology, and chemistry) and in clinical science to the right (e.g., pharmacology, neurology, and radiology) (for details, see González-Márquez et al. 2023). The position of actual paper mill publications obtained by one of us (B.S.) (98 red triangles in A) shows that the large majority are from clinical and not basic science disciplines. The paper mill publications in the 12 o’clock cluster are from cancer research and the cluster in the center from microbiology/virology. **B** Distribution of publications with hospital affiliation only (black) or hospital affiliation and private email address (red). The distribution shows that private email addresses are widespread across fields, forming clusters in some topics but not in others. The private/institutional email address ratio is 894,124/5,800,617 = 15.4%

To gain further insight into paper mill activity, a 2D landscape map was created from 21 million biomedical abstracts using a PubMedBERT large language model analysis of the total PubMed database to produce embeddings of scientific topics (González-Márquezet al. 2024). We then plotted the distribution of actual (true) paper mill publications obtained from a single source (*n* = 98 provided by our paper mill interview partner from China; see Table 2) and projected the email-hospital feature onto a 2D topic landscape (landscape analysis kindly provided by D. González-Márquez; for details, see Fig. 2).

### Validation of detection algorithm

While the first two “AND” rules (email-coauthor or email-hospital) were feasible for early estimates (Studies 1–7), in Studies 8 and 9, we applied a multifactorial tallying rule by either adding or substracting points. If the sum of a given publication was >2 points, it was red-flagged. 

Specifically, one point was counted for each of the following criteria:

- Private email of corresponding author
- Hospital affiliation
- Suspicious country (defined by 20+ retractions because of “Paper Mill” label in retractionwatch database; they were China [2463], Russia [63], Iran [47], India [41], Pakistan [24], Iraq [22], Saudi Arabia [20])
- Publication output of institution: ≤ 200 in all years

-   A point is subtracted, if

-  Number of co-authors  < 1 or > 15
- Publication output of institution (all years): > 1000 publications

This rule was then applied to a sample of *n* = 400 true fake publications as identified by fake gene sequences, text or image plagiarism, and/or retraction (https://retractionwatch.com*)*.

(i) *n* = 100 retractions (using the criterion “paper mill”) (http://retractiondatabase.org/RetractionSearch.aspx?)
(ii) *n* = 100 tadpole-like image plagiarisms (https://docs.google.com/spreadsheets/d/1KXqTAyl4j-jVorFPMD2XRpr76LcIKJ0CVyIvRj0exYQ/edit#gid=0)
(iii) *n* = 100 fake gene sequences; https://dbrech.irit.fr/pls/apex/f?p=9999:28
(iv) *n* = 100 retractions from *Journal of Cellular Biochemistry* (Behl 2021).

The control set of *n* = 400 (presumably) non-fake publications was sampled by randomly selected publications from the years 2000 and 2010, when the use of fake publishing was much smaller (van Noorden 2023). These control publications were obtained by searching for the key words of each fake publication in PubMed and selecting the first item listed. A limitation of this method is that one cannot be certain that each control publication is indeed not fake, though in those years the probability was deemed low.

### Estimating RFP and true fake rates

To estimate the biomedical RFP and true fake rate, in study 9 the validated multifactorial tallying rule was applied to published studies from 10 journals for the year 2020 (*n* = 1,000) and a random key word search (selecting the first reference listed in PubMed) for the year 2023 (*n* = 1,000). The true fake rate in 2023 was estimated using Bayes’ rule. This rule is based on the theorem developed by Thomas Bayes in 1763 (*An Essay Towards Solving a Problem in the Doctrine of Chances*), which describes the probability that an event occurs as a function of a specific condition (conditional probability). Bayes’ theorem is also known as a formula to specify the probability of “causes.”

Finally, we studied > 1,000 websites retrieved from Google and Baidu that advertised various editing services (search terms: “SCI publication or editing service”, “essay writing service”, “journal writing service”, “SCI ghost writing”) and recorded an interview with a paper mill manager (see Table 2).

## Results

### Exploration of fake indicators

**Fig. 3:**
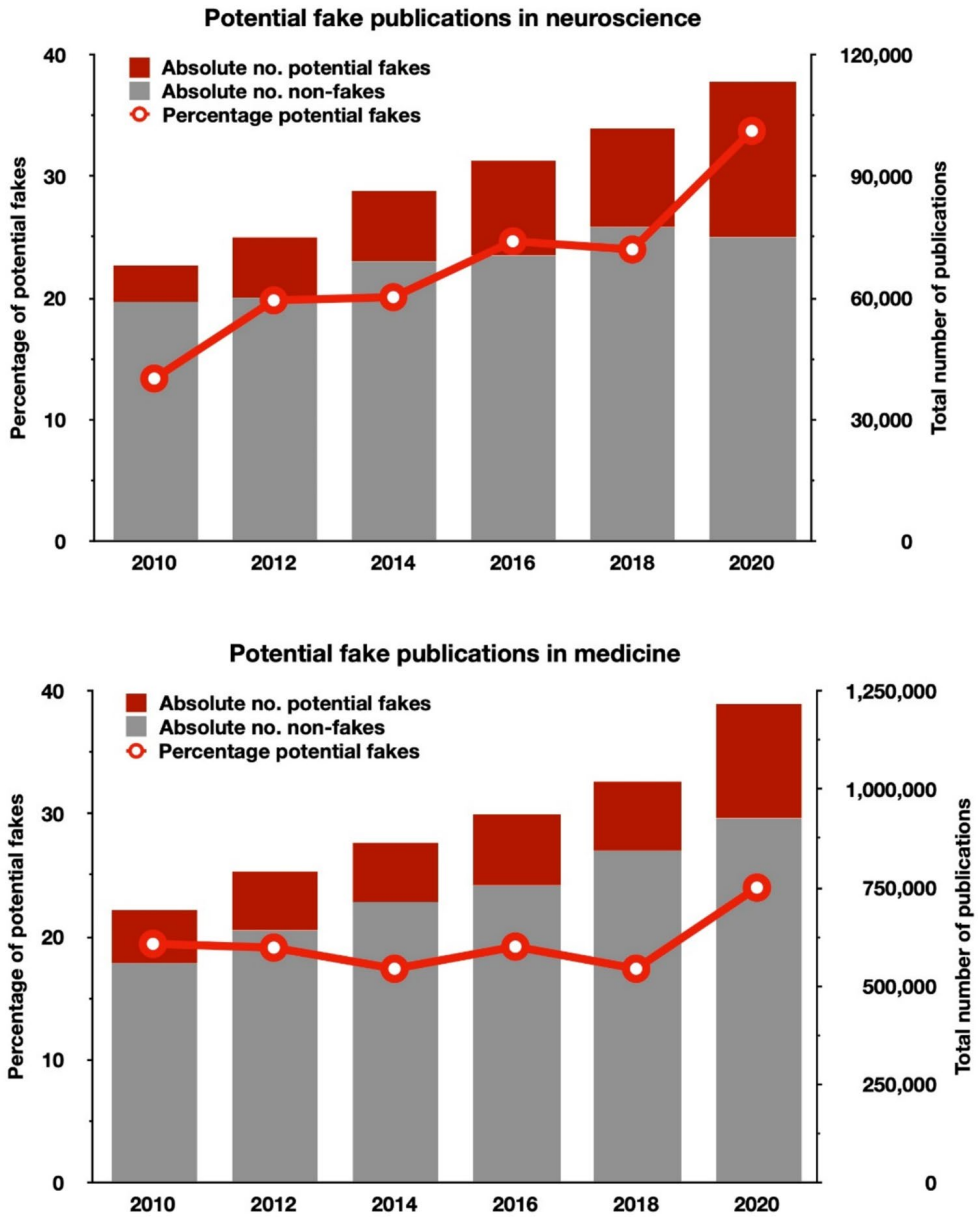
The rise of red-flagged fake publications (RFPs). Number of publications and percentage of red-flagged fake publications in medicine (top) and neuroscience (bottom) (Study 3). The red line (y1-axis) shows the percentage of red-flagged publications per year; the grey bars (y2-axis) show the estimates of non-fakes, and the red bars show those of potentially fake publications, extrapolated to overall articles published in journals of the Neuroscience Peer Review Consortium and in medicine (selected from scimagojr.com). Note the relatively slow increase in potential fakes in medicine and the rapid rise in neuroscience

We searched for indicators that can be determined easily, quickly, and reliably by an editor on the basis of a submitted manuscript or publication alone. Our exploration phase (Studies 1–6) was guided by three hypotheses:

- **Hypothesis 1**: Authors of fake publications are unwilling to answer quality check surveys or provide original data. In Study 1, *n* = 215 neurology articles were manually inspected by an experienced editor; 20.5% (*n* = 44) were deemed suspicious. A questionnaire was sent to the authors and, for control, to 48 authors of unsuspicious papers with questions that authors of fake papers might be reluctant to answer (e.g., “Are you willing to provide original data if so asked?” [only 1 author of 44 suspicious articles responded, compared with 46 of the 48 authors of the unsuspicious articles] and “Did you engage a professional agency to help write your paper?” [none did]; see Table 3). Despite repeated reminders with a warning that failure to reply—or replying inadequately—could trigger retraction, the response rate among suspected authors was only 45.4% (20/44) compared with 95.8% (46/48) for the control group.

- **Hypothesis 2**: Because paper mills are on the rise (Else and Van Noorden 2021), indicators uncovered in Study 1, if valid, should also increase each year. Study 2 analyzed the frequency of these indicators in five randomly chosen neuroscience journals, expanded in Study 3 to a larger sample of articles from those five neuroscience journals and an additional five medical journals every 2 years in the period of biannually 2010–2020. The results show a rapid growth of RFPs over time in neuroscience (13.4 to 33.7%) and a somewhat smaller and more recent increase in medicine (19.4 to 24%) (Fig. 3). One reason for the greater rise of neuroscience RFPs may be that fake experiments in basic medical science (biochemical, in vitro, and in vivo studies) are easier to generate because they do not require clinical trial ethics approval by regulatory authorities.

- **Hypothesis 3**: Because it is easier for paper mills to market their papers to journals with lower journal impact factors (JIF; range 1–6), our indicators, if valid, should also occur more frequently in such journals. Study 4 tested an even larger sample of randomly selected journals of the Neuroscience Peer Review Consortium that red-flagged 366/3,500 (10.5%) RFPs. In Study 5, we counted RFPs in 10 randomly chosen biomedical journals, where the 2020 rate was 23.8% in journals with lower IF; this was topped only by open access (OA) Frontiers journals in Study 6 (112/300 = 40.3%).

### Validation of fake indicators

To explore which indicators could best identify potential fakes and true fakes, we computed sensitivity/false-alarm rates by comparing a sample of known fakes (*n* = 400) with a sample of presumed non-fakes (*n* = 400) (Study 8). Using 2000/2010 publications archived in PubMed as controls, the email-hospital rule had a 85% sensitivity for red flagging publications and a false-alarm rate of 1% (2000) or 8% (2010). But when using the multifactorial tallying rule, the detection sensitivity was 94% (= 6% false negatives/missed fakes) with only 11.5% false alarms, respectively.

Note that classification rules red-flag likely fakes and not proven (true) ones. Nevertheless, it serves as a reliable tool to red-flag scientific reports for further analysis and is a rational basis for estimating the upper value of fake publishing. Detecting fake papers with other indicators (e.g., image duplications and text plagiarism) and in disciplines outside of biomedicine is expected to reveal different results.

The value of the simple email-hospital rule is confirmed by the similarity of a 2D publishing landscape showing that 98 paper mill publications were mostly in clinical science (right half of the landscape) and not in basic science disciplines (left half, Fig. 2A). They were clustered in certain fields (cancer, virology) but not in others (for details regarding the different academic disciplines, see González-Márquez et al. 2024). This clustering was also observed in the total abstract corpus, where the combination of private email address and hospital affiliation (red, Fig. 2B) also formed clusters in certain topics (e.g., cancer, virology, and cardiology) more frequently than in basic biomedical disciplines (e.g., bioinformatics, immunology). Interestingly, the private/institutional ratio of all private email addresses of the corresponding authors in the total PubMed database was 894,124/5,800,617 (= 15.4%), and of the list of 98 DOIs the paper mill provided, 96 were detected by our indicators.

### Estimating the incidence of potentially fake publications

When estimating the global RFP incidence with the email-hospital rule (Table 4), most red-flagged articles came from China (42.3%) and India (33%). Within-country percentages of RFPs vary considerably and are highest for Russia (66.7%), India (54.2%), China (38.7%), Iran (29.6%), and Turkey (20.8%). Overall, the estimated RFP rates are 14.9% in 2020 and 16.3% in 2023, close to the 20.4% of our author questionnaire in Study 1 and the 15.4% private/institutional ratio of all email addresses in PubMed (see Fig. 2 legend).

**Table 4:** Countries’ contribution of potential fake publications (RFP)

	Within country			Between countries		
Country	2020	2023	Total	2020	2023	Total
China	40.5%	33.3%	38.7%	69.8%	17.2%	42.3%
India	28.6%	66.9%	54.2%	12.1%	52.2%	33.0%
Iran	20.0%	42.1%	29.6%	3.4%	4.9%	4.2%
Turkey	22.2%	20.0%	20.8%	2.7%	4.3%	3.5%
Russia	66.7%	*	66.7%	5.4%	*	5.4%
Japan	0.0%	11.6%	7.0%	0.0%	3.1%	1.6%
UK	0.0%	5.4%	4.5%	0.0%	2.5%	1.3%
Spain	6.9%	4.0%	5.6%	1.3%	0.6%	1.0%
South Korea	7.1%	4.6%	5.2%	0.7%	1.2%	1.0%
USA	0.0%	1.0%	0.4%	0.0%	1.2%	0.6%
Germany	0.0%	7.1%	3.9%	0.0%	0.6%	0.3%
Italy	6.3%	0.0%	2.3%	0.7%	0.0%	0.3%
Brazil	2.7%	0.0%	1.6%	0.7%	0.0%	0.3%
Canada	0.0%	0.0%	0.0%	0.0%	0.0%	0.0%
Mexico	0.0%	0.0%	0.0%	0.0%	0.0%	0.0%
France	0.0%	0.0%	0.0%	0.0%	0.0%	0.0%
Netherlands	0.0%	0.0%	0.0%	0.0%	0.0%	0.0%

Estimated rate of red-flagged publications (RFP) within individual countries and between country in the PubMed database. Only countries with > 1% of publication output were considered in the respective year (*Russia fell below 1% of global publishing in 2023)

### Estimating the incidence of true fake publications

Using Bayes’ rule, we estimated the number of *true fake* publications as of 2023. The rate (probability, *p*) of positive results is 0.163, and the sensitivity and false-alarm rates are 0.94 and 0.115, respectively. We use *D* (data) for a positive test result (a red flag), *H* (hypothesis) for a true fake, and –*H* for no fake.

Because

$$p(D) = p(D|H)p(H) + p(D|-H)p(-H),$$

we get:

$$.163 = .94p(H) + .115p(-H) = .94p(H) + .115[(1-p(H)],$$

$$.163 - .115 = (.94 - .115)p(H) = .048 = .825p(H), and$$

$$p(H) = .048/.825 = .058.$$

That is, the estimated rate of true fake publications is 5.8%. This figure is, of course, dependent on the specific indicators (rules) used, and the databases surveyed, in this article.

In the same way, the positive predictive value of a red flag can be estimated as *p*(*H*|*D*) =.335, or 33.5%. That is, in about one third of cases the red flag is correct (i.e., a true fake).

Given the 2023 global publication output of 1.86 million biomedical publications in 2023 (Scimago) and the estimated 5.8% true fake rate, the annual number of fake articles is around 107,800 for that year. This is about 19 times higher than the 5671 retractions in biomedicine (not counting 622 “honest mistakes”; see https://retractiondatabase.org/RetractionSearch.aspx*)*. Furthermore, assuming conservatively that the number of true fake publications increased by a factor of 4 from 2010 to 2023 (see the increasing red flag rate in Fig. 3), during this 14-year period, the contamination of the permanent scientific record by true fake publications is at least in the order of 760,000, not including those missed by our detection method (such as image plagiarisms), publications listed in PubMed prior to 2010, or “predatory” journals.

Assuming an average $10,000 price tag for a fake publication, the roughly estimated annual revenue of paper mills in biomedicine is in the order of $1 billion in 2023 (3 billion if extrapolating the rate to all science disciplines). This sum includes neither revenue from publications in journals not ranked by Scimago or predatory journals nor the associated publishers’ subscriptions and/or open access publishing charges. Although the incidence of true fake publications is smaller than our RFP estimate, given the 94% detection rate with a rate of 11.5% false alarms and 6% false negatives, the number of fake publications is worrying, and it is on the rise.

### Qualitative analysis of paper mill strategies

More than 1,000 paper mills openly advertise their services on Google and Baidu to “help prepare” academic term papers, dissertations, and articles intended for SCI publications. Most paper mills are located in China, India, UK, and USA; some are multinational. They use sophisticated, state-of-the-art AI-supported text generation, data and statistical manipulation and fabrication technologies; image and text pirating; and gift or purchased authorships. Paper mills fully prepare—and some guarantee—publication in an SCI journal and charge hefty fees ($1,000–$25,000; in Russia: $5,000) (Chawla 2022) depending on the specific services ordered (topic, impact factor of target journal, with/without fake “experimentation”).

An unsolicited meeting with a paper mill provided a rare and authentic inside view of their business practices (Table 2). Paper mills employ science graduates, academicians, and (sometimes naïve) scientific consultants for editorial help who work in countries with high English aptitude (UK, USA, India). They also offer “rewards” (bribes) to editors for publishing their fabrications (Table 1). We know of at least 12 such cases (two reported by editors, 10 acknowledged by an academic publisher who asked not to be identified). Editors were bribed with payment for each publication and with a “citation booster,” whereby paper mills offered to cite the editor’s “friendly” journal in their other fake publications. Although we do not know how many editors have received or accepted such bribes, it is an unprecedented and disturbing form of fraud for profit in scholarly publishing.

## Discussion

The dramatic rise of fake science publishing is driven by an unscrupulously corrupt—and increasingly successful—paper mill industry responsible for an estimated 107,800 true fake articles annually as of 2023. This is a number considerably higher than current estimates (Table 1) and 19 times higher than the number of retractions in biomedicine that year. Our 5.8% estimate of true fakes in 2023 is much higher than the 2011 estimate of 0.1% fake publications in China (Hu and Wu 2013) and the 1% listed in Table 1, but similar to the 5–10% reported in a pharmacology (Seifert 2021) and a cancer journal (Heck et al. 2021), and below the 21–32% honorary, guest, and ghost authorships in biomedicine (Else 2021; Flanagin et al. 1998; Wislar et al. 2011). By now, paper mills are a billion-dollar global industry, magnitudes higher than the $4.5 million monetary value estimated in 2011 (Hu and Wu 2013).

Our analysis confirms the existence, continuous growth, and notable scope of fake publishing, with most red-flagged publications coming from Chinese hospitals (69.8% in 2020). Interestingly, a sleuth who wanted not to be named reported that ~30% of manuscripts containing images are fraudulent which can be very effectively determined by manual checking (van Diest et al. 2025).

That our 98 paper mill publications were clustered in certain subfields of biomedicine (e.g. non-clinical cancer and cardiovascular studies) matches our observation of the co-localization of “private email address” and “hospital affiliation” in the total PubMed database. As the 2D topic landscape distribution confirms, paper mills specialize in certain topics, and they use private or fake institutional email addresses when submitting manuscripts to, and communicating with, journal editors.

Our study has some limitations: The journal and topic selection limits it to the field of biomedicine, and given 1.86 million publications in this field in 2023 alone, our sample of 17,120 screened publications across several years is several magnitudes smaller. While the tallying method has a low 11.5 % false alarm rate (“wrong accusations”) and only 6% false negatives (“missed fakes”), it does not allow an unambiguous judgment for a given paper. Nevertheless, we were able to estimate that the probability of a true fake given a red flag is 33.5%. Another limitation of our study is that our red-flagging method does not directly translate to other science disciplines such as computer science or chemistry, an issue that needs to be addressed by others.

The 5.8% estimate of true fakes is between the 2% retractions (proven fakes) that accumulated for >10 years and the 30% range of the number of image manipulations, respectively. It is therefore the most reasonable approximation at this time in the field of biomedicine. Whatever the exact number of fakes might be - and we expect it to be higher -, we conclude that the fake publishing crisis is too large to ignore and it is the largest scientific scam of all times.

The rapid rise of the fake publishing industry is driven by incentive systems forcing scientists to publish in SCI journals, who then hire paper mills’ fraudulent services at $1,000 to $25,000 per publication (Hu and Wu 2013; Tian et al (2016). Academic publishers have acknowledged the problem (COPE and STM Committee on Publication Ethics 2022, Declaration “United2 Act” 2023) and started implementing detection tools (Else 2022). Time will tell how effective such activities are and how diligent and transparent the academic publishers’ effort will be to detect fake manuscripts and remove those published from the permanent scientific record. Chinese authorities, albeit aware of the situation for years (Cyranoski 2018), have not sufficiently resolved the problem yet. It remains to be seen what the practical outcome will be of their recent investigations asking authors of 17,000 retractions to explain themselves (Mallapaty 2024) and sleuths’ call to “stamp out paper mills” (Abalkina et al. 2025); recent actions by the Chinese superior court are an encouraging signal (Mallapaty 2025).

If publication output is viewed as an index of world leadership in science, then paper mills contribute to reaching this goal. Applying this metric, China has outcompeted the USA with ever more publications since 2019. We submit that reducing the publication pressure especially on hospital-affiliated scientists (mostly medical doctors) will significantly help to prevent contamination of the scientific record.

Paper mills also feed on the rising worldwide administrative practice of evaluating researchers by the publish-or-perish criteria of counting papers and JIF ranking metrics as a surrogate for evaluating research quality and content (Van Dalen and Henkens 2012; Candal-Pedreira 2022). Beyond the fact that gaming the impact factor through corruption dilutes its value as a metric of scientific achievement, a recent bibliometric experiment exposes the absurdity of such metrics: A fictitious author (Larry, the cat) achieves an h-index of 12 within only 1 year (Wilcox 2024, reporting a blog by Reese Richardson).

Fake publishing is also a growing risk for medical practice. For example, Byrne et al. showed that among 712 problematic papers that were cited >17,000 times, about one quarter may misinform future development of human therapies (Park et al. 2022). Preclinical studies at biotech company Amgen could replicate the results of only 6 out of 53 “landmark” articles, and at Bayer, only 14 of 67 were replicable in oncology, women’s health, and cardiovascular medicine. This reproducibility crisis—to which fake publications contribute—slows down the development of life-saving therapies with an estimated financial loss of $28 billion annually by the pharmaceutical industry (Gigerenzer 2018). Yet another example of how scientific fraud can affect medical practice is reported by Avenell et al. (2019). After assessing the citations of 12 retracted clinical trial reports in 68 systematic reviews, meta-analyses, guidelines, and clinical trials, they concluded that 13 of the latter would likely have to change their conclusions without the data from the retracted publications. Mass retractions, especially in the last 2 years, have not only awakened academic publishers, funders, and the scientific community to the fact that fake publications are of growing concern (Mallapaty 2024).

Our red-flagging and tallying methods may help combat this problem, but it is important to keep in mind that our indicators are not legal proof of a given manuscript or publication being unequivocally fake. However, at the peer-review stage it is the authors’-not the editors’- burden of proof to demonstrate that their work can be trusted. But once their manuscripts are published, the burden of proof is with the publisher. Whether this type of scientific misconduct is a conspiracy to commit injurious falsehood or a crime is for others to decide.

Industrial-style fake science publishing in the biomedical sciences damages our societies, wasting financial resources, slowing down medical progress, and possibly endangering lives. The full damage done is still unknown, and a realistic impact assessment of fake publishing is not yet available. The emergence of large language models (artificial intelligence) amplifies the problem, as they lower the production cost of fake papers and, moreover, are being trained on data that include fakes. Our easy-to-apply indicators can be a first step to help stave off fake publishing.

Halting the massive proliferation of fake publications requires a timely response from the scientific community and the publishers. Because the business model of the academic publishing industry is to generate revenue from scientific publications, fake publishing makes publishers vulnerable to disruptive change (Booth et al. 2023). While each published fake increases a publishers’ revenue at relatively low costs in special issues published online in open source, it also carries the risk of knowledge collapse when these fakes systematically distort knowledge.

The following measures should therefore be implemented to reduce the science publishing crisis (Sabel 2024):

- The academic community should consider revising its practice to judge scientists’ productivity mostly (or solely) on surrogate quantitative criteria of a publish-or-perish culture (impact factors, publication numbers, h-index, etc.). We need fewer articles of better quality and should evaluate the content, quality, and relevance of research (Van Dalen and Henkens 2012). The European Research Council (ERC) has already taken a first step by asking researchers to refrain from listing JIF in their applications, consistent with the San Francisco Declaration on Research Assessment (DORA 2012).
- Raise awareness of the publishing crisis among science communities, publishers, funders, industry, foundations, and governments.
- Teach editors and scientists how to understand and avoid publishing fraud.
- Keep an eye on, and sanction, editors who have been successfully bribed by paper mills.
- Develop and improve easy-to-apply methods for detecting fakes and train editors and reviewers how to use these.
- Learned societies, funding agencies, and governmental bodies should consider sanctioning journals containing fake publications and their publishers, e.g., by public blacklists or reducing/stopping funding of academic publishing charges (APCs).
- Post-publication review (e.g., PubPeer) may help reduce integrity concerns.
- Transparent and full disclosure of third-party service providers (Teixeira da Silva et al. 2024).
- In addition to the journal impact factors, consider introducing a *journal fake factor*, defined as the proportion of published fakes among all publications in the last 2 years. In our study, the average journal fake factor would be at around 5.8%.

Until the industry-style production of fake articles is largely eradicated, we will have to learn to live with the collateral damage in the biomedical sciences. Public health information will be less accurate or (intentionally) misleading, and presumably effective and safe therapies may not deliver what is promised. What is more, we face the risk that increasingly more people lose their trust in the integrity of science. Simple detection rules for fake publications, as proposed here, or more complex automated methods (with or without AI) can help prevent further damage to the permanent scientific record and enable the retraction of fake publications at scale. We propose a call to action to restore the integrity of our global knowledge base not only in biomedicine but also in other scientific and technological fields.

## Data Availability

All source data for this work (or generated in this study) are available upon reasonable request.
